
JOURNAL INFORMATION
==============================
NLM Title Abbreviation: Wirtschaftsdienst
Journal ID: Wirtschaftsdienst
NLM Journal ID: 101086612

Wirtschaftsdienst (Hamburg, Germany : 1949)

ISSN: 0043-6275

ARTICLE INFORMATION
==============================
PMCID: PMC7813617
PMID: 33487769
DOI: 10.1007/s10273-021-2819-3
Article ID: 2819
Article version: 1
Subjects: Zeitgespräch

Verteilungsfolgen der Corona-Pandemie: Staatliche Sicherungssysteme und Hilfsmaßnahmen stabilisieren soziales Gefüge

Beznoska Martin beznoska@iwkoeln.de
1
Niehues Judith niehues@iwkoeln.de
2
Stockhausen Maximilian stockhausen@iwkoeln.de
3
1 grid.473597.b 0000 0000 9854 4639 Steuer- und Finanzpolitik, Institut der deutschen Wirtschaft Köln e.V., Konrad-Adenauer-Ufer 21, 50668 Köln, Deutschland
2 grid.473597.b 0000 0000 9854 4639 Methodenentwicklung, Institut der deutschen Wirtschaft Köln e.V., Konrad-Adenauer-Ufer 21, 50668 Köln, Deutschland
3 grid.473597.b 0000 0000 9854 4639 öffentliche Finanzen, soziale Sicherung u. Verteilung, Institut der deutschen Wirtschaft Köln e.V., Konrad-Adenauer-Ufer 21, 50668 Köln, Deutschland
Electronic publication date: 2021 Jan 19
Print publication date: 2021
Volume: 101
Issue: 1
First page: 17
Last page: 21
Copyright: © Der/die Autor(en) 2021
License: Open Access: Dieser Artikel wird unter der Creative Commons Namensnennung 4.0 International Lizenz (https://creativecommons.org/licenses/by/4.0/deed.de) veröffentlicht.
Open Access wird durch die ZBW — Leibniz-Informationszentrum Wirtschaft gefördert.
License URL: https://creativecommons.org/licenses/by/4.0/

issue-copyright-statement: © The Author(s) 2021

==============================

Die Corona-Pandemie hat das Jahr 2020 maßgeblich bestimmt und tiefe Spuren in Wirtschaft und Gesellschaft hinterlassen. Mit zunehmender Krisendauer rückt die Frage nach den Folgen der Corona-Pandemie auf die soziale Ungleichheit ins öffentliche Interesse. Darüber wie sich die Verluste der Corona-Krise in der Bevölkerung verteilen und inwiefern staatliche Hilfsmaßnahmen die negativen Folgen abfedern konnten, ist jedoch wenig bekannt. So fehlen aktuelle Mikrodaten zu den krisenbedingten Einkommensveränderungen oder diese werden voraussichtlich erst mit Verzögerung der (Fach-)Öffentlichkeit zur Verfügung stehen.

Zudem ist das Infektionsgeschehen nicht beendet und weitere Lockdowns mit all ihren negativen wirtschaftlichen Konsequenzen sind denkbar.

Was lässt sich aus bisherigen Studien und Umfragen über die Verteilung der finanziellen Lasten der Corona-Pandemie in Deutschland ableiten? Zu Beginn der Pandemie waren vor allem weniger gut ausgebildete Menschen von Kurzarbeit und entsprechenden Einkommenseinbußen betroffen, wie Auswertungen von Schröder et al. (2020) auf Basis der SOEP-CoV Sonderbefragung zu den Auswirkungen der Corona-Pandemie zeigen. Besser Ausgebildete konnten hingegen häufiger ihre Arbeitszeit reduzieren oder auch im Homeoffice arbeiten und waren damit seltener von Kurzarbeit betroffen. Trotz dieser Unterschiede fiel jedoch der Anteil der Personen mit Einkommensverlusten unter den Personen mit geringem und hohem Bildungsabschluss relativ ähnlich aus und lag jeweils bei knapp 20 %. Auf Haushaltsebene zeigt sich zudem, dass beispielsweise der Anteil der Kurzarbeitenden im unteren und oberen Einkommensbereich der Haushalte mit jeweils knapp 20 % auch hier auf einem sehr ähnlichen Niveau lag. Ähnliches galt für die Verteilung der Einkommensverluste.

Darüber hinaus zeigen sich Unterschiede in der Betroffenheit verschiedener Erwerbsgruppen: Selbstständige waren besonders häufig und stark von der Krise betroffen. In den Monaten April und Mai 2020 gaben rund 60 % der Selbstständigen an, Einkommensverluste erlitten zu haben, gegenüber 15 % der abhängig Beschäftigten (Kritikos et al., 2020). Zu ähnlichen Ergebnissen kommen auch Untersuchungen aus dem Erwerbstätigenpanel des Wirtschafts- und Sozialwissenschaftlichen Instituts (WSI) für April und Juni. Dort gaben rund 32 % aller Erwerbstätigen an, einen Einkommensverlust erlitten zu haben, während es unter den Selbstständigen und Unternehmer:innen rund 56 % waren. Ebenfalls waren Leiharbeiter:innen und geringfügig Beschäftigte im Durchschnitt deutlich stärker von Einkommensverlusten betroffen als beispielsweise Beamte. Die Befragungsergebnisse zeigen zudem, dass besonders häufig Erwerbstätige mit vergleichsweise geringem Einkommen Verluste während der Corona-Krise erlitten haben (Hövermann, 2020).

Allerdings beschränken sich die bisherigen Analysen entweder auf die Betrachtung einzelner Bevölkerungsgruppen, beispielsweise auf die Gruppe der Erwerbsfähigen wie in der WSI-Befragung, oder sie bilden nur die Frühphase der Corona-Pandemie ab. Eine umfassende Betrachtung der Auswirkungen auf die Einkommensverteilung, die sowohl die Gesamtbevölkerung als auch die Wirkung unterschiedlicher finanzieller Hilfsmaßnahmen der Bundesregierung zur Einkommensstabilisierung im gesamten Jahresverlauf in den Blick nimmt, fehlte bislang. Aus diesem Grund hat das Institut der deutschen Wirtschaft (IW) im August zunächst eine Online-Befragung in Auftrag gegeben, in der 1.202 Personen zu ihren Einkommensveränderungen infolge der Pandemie befragt wurden sowie bezüglich ihrer bisherigen und noch zu erwartenden Betroffenheit von Kurzarbeit.[1] Wesentliche sozio-ökonomische Charakteristika wurden inhaltsgleich zu den Informationen im SOEP (Sozio-oekonomisches Panel) erhoben, um beide Datensätze in einem zweiten Schritt miteinander verknüpfen zu können. Dafür wurde das SOEP bis zum Jahr 2019 fortgeschrieben und anschließend wurden Personen aus der IW-Umfrage mithilfe des Mahalanobis Distance Matching an statistische Zwillinge im SOEP herangespielt.[2] Die Informationen aus den gematchten Beobachtungen der IW-Umfrage wurden dann dazu verwendet, um in einer Mikrosimulation krisenbedingte Einkommens- und Statusveränderungen der SOEP-Population zu simulieren. Die sich daraus neu ergebenden Steuern und Abgaben wurden mithilfe des Steuer-, Abgaben- und Transfer-Mikrosimulationsmodells (STATS) des IW berechnet. Finanzielle Hilfen zur Bekämpfung der negativen Folgen der Lockdown-Maßnahmen wurden ebenfalls modelliert, insofern sie bis Ende November beschlossen waren. Dazu zählen die Erweiterung beim Kurzarbeitergeld, der Kinderbonus, der vereinfachte Zugang zur Grundsicherung und die Erhöhung des Alleinerziehendenentlastungsbetrags. Sämtliche Hilfszahlungen an Selbstständige wie z. B. Soforthilfen oder die Überbrückungshilfe I, die zwischen Juni und August galt, sind nicht explizit in der Simulation berücksichtigt. Jedoch ist es wahrscheinlich, dass die im August befragten Selbstständigen bei den subjektiven Einschätzungen zu den Auswirkungen der Corona-Pandemie auf ihr Bruttogewinneinkommen zumindest teilweise die Hilfszahlungen mitberücksichtigt haben.

1 Eine Auswertung zum Kinderbonus sowie weitere Erläuterungen zur Befragung finden sich in Beznoska et al. (2020a).

2 Ausführliche Erläuterungen zum Matching-Verfahren finden sich in Beznoska et al. (2020b), auf die sich die folgenden Ausführungen maßgeblich beziehen.

Abbildung 1

**Veränderung der Haushaltseinkommen durch die Corona-Pandemie**

Dezile nach Verteilung der Nettoäquivalenzeinkommen im Jahr 2019 (neue OECD-Skala), Veränderungen gegenüber 2019 in %

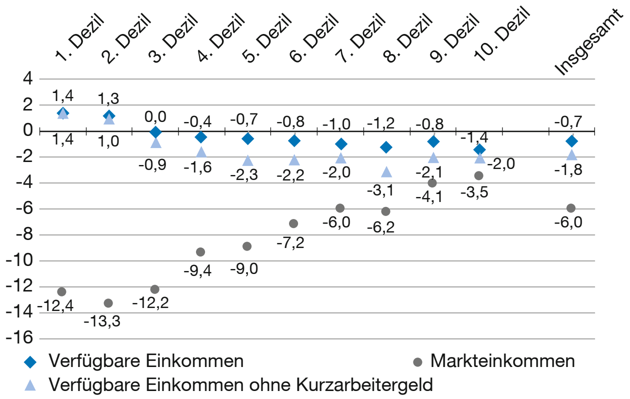

Quelle: Beznoska et al. (2020b).

Die Ergebnisse der aus SOEP und IW-Umfrage kombinierten Datenbasis zeigen einerseits, dass die Einkommensverluste in der Bevölkerung breit verteilt sind und die Einkommen insgesamt gesunken sind. Die Markteinkommen gingen im Krisenjahr im Durchschnitt um 6 % zurück (vgl. Abbildung 1). Die größten relativen Veränderungen sind dabei in der unteren Hälfte der Einkommensverteilung zu beobachten. Im 2. Dezil liegt der durchschnittliche Rückgang der Markteinkommen bei rund 13 % währenddessen er im 9. Dezil bei durchschnittlich 4 % liegt. Im 5. Dezil beträgt der Rückgang rund 9 % Die Dezile wurden hierbei über die Verteilung der aufsteigend sortierten Nettoäquivalenzeinkommen des Jahres 2019 gebildet.

Bei Betrachtung der verfügbaren Haushaltseinkommen zeigt sich der ausgleichende Effekt der sozialen Sicherungssysteme, die in der Krise um zusätzliche temporäre Hilfszahlungen wie etwa den Kinderbonus verstärkt wurden. In der Folge reduzieren sich die relativen Verluste in der Einkommensmitte und am unteren Ende der Einkommensverteilung. Durch einen vereinfachten Zugang zur Grundsicherung, die Erhöhung des Steuerfreibetrags für Alleinerziehende oder die Ausweitung von Wohngeldleistungen und Kinderzuschlag – die unabhängig von der Corona-Krise in diesem Jahr umgesetzt wurden – ergibt sich für das 1. und 2. Einkommensdezil sogar ein leichtes nominales Plus bei den verfügbaren Haushaltseinkommen. Einschränkend muss dazu gesagt werden, dass dieses Ergebnis unter der Annahme steht, dass die Sozialleistungen zu 100 % in Anspruch genommen werden.

Aber auch die Einkommensmitte profitiert in der Corona-Krise von sozialstaatlichen Umverteilungs- und Hilfsmaßnahmen und hierbei insbesondere vom Kurzarbeitergeld. Reduzieren sich die Markteinkommen im 5. Dezil um durchschnittlich 9 %, fällt der Verlust bei den verfügbaren Einkommen mit durchschnittlich 0,7 % erheblich geringer aus. Ohne das Kurzarbeitergeld hätte der Verlust bei rund 2 % gelegen. Mit Blick auf die oberen Einkommensgruppen zeigt sich, dass die Besserverdienenden in der Krise zwar die relativ geringsten Verluste in den Markteinkommen zu verzeichnen haben, aber die prozentual größten Verluste in den verfügbaren Einkommen, da Hilfsmaßnahmen in diesem Einkommensbereich eine relativ zum höheren Einkommen geringere Bedeutung haben. Ebenso spielen hier auch andere Einkommensarten wie Kapitaleinkommen oder Einkommen aus Vermietung und Verpachtung eine Rolle, die stabilisierend auf das Gesamteinkommen eines Haushalts gewirkt haben können.

Zu den von der Corona-Pandemie besonders stark betroffenen Erwerbsgruppen zählen neben Selbstständigen, die im Durchschnitt rund 3 % ihrer verfügbaren Einkommen verloren haben, geringfügig Beschäftigte. Am stärksten betroffen sind Selbstständige im mittleren und oberen Einkommensbereich. Dort fallen die Verluste mit durchschnittlich bis zu 12 % der verfügbaren Haushaltseinkommen am höchsten aus. Hingegen verzeichnen geringfügig Beschäftigte im untersten Einkommensdezil mit durchschnittlich rund 5 % der verfügbaren Haushaltseinkommen die höchsten Ausfälle in ihrer Erwerbsgruppe. Beide Erwerbsgruppen haben in der Krise in aller Regel keinen Anspruch auf Kurzarbeitergeld, was eine Erklärung für die größeren Einkommensrückgänge darstellen dürfte. Allerdings sind Selbstständige häufiger im mittleren bis oberen Einkommensbereich verortet und Verluste können häufiger über den Haushaltskontext aufgefangen werden.

Anders als oft vermutet, fallen die Einkommensveränderungen zwischen den Geschlechtern bei Betrachtung der verfügbaren Haushaltseinkommen sehr ähnlich aus, und auch Alleinerziehende sind diesbezüglich nicht auffällig stärker betroffen als andere Familientypen. Vielmehr fallen die Einkommensverluste von Paaren mit Kindern in vielen Fällen am höchsten aus, was auf eine reduzierte Arbeitszeit zum Zwecke der Kinderbetreuung infolge von Schul- und Kita-Schließungen zurückzuführen sein könnte. Darüber hinaus sind Personen im Alter zwischen 40 und 49 Jahren im Durchschnitt am stärksten von Einkommensverlusten betroffen, währenddessen Personen über 70 Jahre aufgrund gestiegener Altersrenten auch im Krisenjahr Einkommenszuwächse verzeichnen können.[3] Grundsätzlich muss angemerkt werden, dass die Simulationsanalyse nur unmittel-

3 Da sich für das Jahr 2021 bereits ein geringerer Zuwachs bei der gesetzlichen Rente andeutet (gegenüber prognostizierten höheren Zuwächsen bei den Bruttoerwerbseinkommen), verschieben sich die finanziellen Auswirkungen der Corona-Krise für Ruheständler teilweise auf das kommende Jahr. Aufgrund der Rentengarantie ist ein nominaler Rückgang der Renten jedoch gesetzlich ausgeschlossen.

Abbildung 2

**Veränderung der Verteilung von Markt- und Nettoeinkommen durch die Corona-Pandemie**

Gini-Koeffizient der bedarfsgewichteten Haushaltseinkommen (neue OECD-Skala)

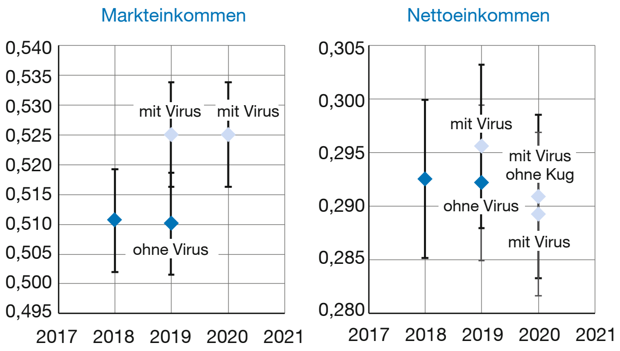

Anmerkungen: Kug = Kurzarbeitergeld; Whiskers stellen 95 %-Konfidenzintervalle dar (Bootstrap-Verfahren, n=100); der Zusatz mit/ohne Virus zeigt an, ob coronabedingte Einkommensänderungen modelliert wurden oder nicht.

Quelle: Beznoska et al. (2020b).

bare monetäre Auswirkungen der Krise berücksichtigt und nicht-finanzielle Belastungen – beispielsweise durch Betreuungsherausforderungen während der Lockdowns – außen vor bleiben.

All die beschriebenen Einkommensveränderungen führen insgesamt dazu, dass sich die Ungleichheit in den Markteinkommen durch die Krise erhöht, nicht aber die Ungleichheit in den verfügbaren Einkommen (vgl. Abbildung 2). In beiden Fällen beruht die Aussage auf Veränderungen des Gini-Koeffizienten, der Werte zwischen 0 und 1 annehmen kann. Ein Wert näher 1 steht für eine ungleichere Verteilung, während 0 eine Gleichverteilung anzeigt. Der Gini-Koeffizient der bedarfsgewichteten Markteinkommen erhöht sich durch die Corona-Pandemie und die damit verbundenen Einkommensveränderungen von 0,511 im Jahr 2018 auf 0,525 im Jahr 2020. Hingegen liegt der Gini-Koeffizient der bedarfsgewichteten Haushaltsnettoeinkommen nicht nur grundsätzlich auf einem geringeren Niveau als der Gini-Koeffizient der Markteinkommen, sondern geht gemäß den Simulationsergebnissen von rund 0,293 im Jahr 2018 auf 0,289 im Jahr 2020 zurück. Die Veränderungen sind zum Signifikanzniveau von 95 % zwar statistisch nicht voneinander verschieden, legen aber nahe, dass eine krisenbedingte Erhöhung der Ungleichheit in den verfügbaren Haushaltseinkommen eher unwahrscheinlich ist.

Dass dieser Effekt in besonderer Weise auf die ergriffenen Hilfsmaßnahmen zurückzuführen ist, zeigt der Vergleich mit der hypothetischen Situation im Jahr 2019, in der dieselben coronabedingten Einkommensveränderungen angenommen werden, aber die entsprechenden Hilfsmaßnahmen nicht modelliert werden („2019 mit Virus“). In diesem Fall erhöht sich der Gini-Koeffizient der Nettoeinkommen auf rund 0,296 gegenüber ansonsten 0,292 ohne die krisenbedingten Einkommensänderungen („2019 ohne Virus“). Die Verwendung alternativer Ungleichheitsmaße ergibt vergleichbare Ergebnisse bezüglich der Richtung der Ungleichheitsveränderungen (Beznoska et al., 2020b).

## Fazit

Die Corona-Pandemie wird auch in diesem Jahr eine große Herausforderung für Deutschland und die Welt bleiben. Zwar lässt die Bereitstellung von Impfstoffen darauf hoffen, dass die Pandemie langfristig besiegt werden kann. Bis die Pandemie beendet sein wird, ist jedoch ein langer Weg zu gehen. Über die Verteilungsfolgen der Corona-Pandemie, die mit den Lockdown-Einschränkungen einhergehen, ist bislang nur wenig bekannt. In einer Mikrosimulationsstudie haben wir daher betrachtet, welche Veränderungen sich in den Markteinkommen und in den verfügbaren Einkommen der Haushalte coronabedingt ergeben haben.

Auf Grundlage der Kombination von eigenen Umfragedaten und fortgeschriebenen SOEP-Daten ergeben sich zwei zentrale Ergebnisse: So zeigen sich relativ größere Verluste in den Markteinkommen für Bezieher:innen niedriger Einkommen, die jedoch durch effektiv funktionierende sozialstaatliche Sicherungsleistungen abgefedert werden können. Ein Auseinanderdriften der verfügbaren Haushaltseinkommen wird dadurch in der Krise vorerst verhindert. Entgegen der vorherrschenden Meinung ist gemäß der Simulationsanalyse eher nicht damit zu rechnen, dass die soziale Ungleichheit kurzfristig zunimmt. Allerdings bleibt davon unberührt, dass sowohl die durchschnittlichen Markteinkommen als auch die verfügbaren Einkommen krisenbedingt sinken und die Krise somit Wohlstand kostet, und zwar erheblich.

Bezüglich der langfristigen Auswirkungen der Corona-Pandemie wird es darauf ankommen, ob effektive staatliche Hilfen bis zum Ende der Pandemie bereitgestellt werden können, um Insolvenzen wirtschaftlicher Unternehmen weiterhin zu verhindern und Arbeitsplätze dauerhaft zu sichern. Mit längerer Dauer der Pandemie wird es jedoch immer schwieriger, pauschale Hilfen zu leisten, da diese mit hohen Finanzierungskosten einhergehen. Daher sollte sich die Politik in den kommenden Wochen stärker mit der Effizienz staatlicher Hilfsmaßnahmen auseinandersetzen und noch stärker tatsächliche Bedarfe berücksichtigen.

Wenngleich die Simulationsergebnisse unter den gebotenen methodischen Vorbehalten zu interpretieren sind und somit eine erste Abschätzung der allgemeinen Verteilungsfolgen der Corona-Pandemie darstellen, machen sie die Leistungsfähigkeit der sozialen Sicherungssysteme in Deutschland deutlich. Die Robustheit der Ergebnisse wird durch eine Simulationsanalyse von Bruckmeier et al. (2020) bekräftigt, die mit einem anderen methodischen Vorgehen ebenfalls zu dem Ergebnis kommen, dass im Krisenjahr 2020 nicht mit einem Anstieg der Ungleichheit der verfügbaren Einkommen zu rechnen ist. Dabei soll diese statistische Aussage, die nur unmittelbare Einkommensveränderungen in den Blick nimmt, keinesfalls individuelle Notlagen bagatellisieren. Bislang konnten die sozialen Sicherungssysteme sowie die Hilfsmaßnahmen der Bundesregierung jedoch viele Verluste effektiv abfedern und das soziale Gefüge stabilisieren.

Dr. Martin Beznoska ist Referent für Steuer- und Finanzpolitik am Institut der deutschen Wirtschaft in Köln.

Dr. Judith Niehues ist Leiterin der Forschungsgruppe Mikrodaten und Methodenentwicklung des Instituts der deutschen Wirtschaft in Köln.

Dr. Maximilian Stockhausen ist Referent für Verteilungsfragen am Institut der deutschen Wirtschaft in Köln.


Literatur

Beznoska, M., J. Niehues und M. Stockhausen (2020a), Etwa die Hälfte des Kinderbonus soll ausgegeben werden, IW-Kurzbericht, Nr. 92, Köln.
Beznoska, M., J. Niehues und M. Stockhausen (2020b), Stabil durch die Krise? Verteilungsfolgen der Corona-Pandemie — eine Mikrosimulations-analyse, IW-Report, Nr. 65, Köln.
Bruckmeier. K., A. Peichl, M. Popp, J. Wiemers und T. Wollmershäuser (2020), Covid-19-Krise: Für das Jahr 2020 ist mit keinem Anstieg der Einkommensungleichheit in Deutschland zu rechnen, ifo Schnelldienst Digital, Nr. 15, München.
Hövermann, A. (2020), Soziale Lebenslagen, soziale Ungleichheit und Corona — Auswirkungen für Erwerbstätige, WSI Policy Brief, Nr. 44, Düsseldorf.
Kritikos, A. S., D. Graeber und J. Seebauer (2020), Corona-Pandemie wird zur Krise für Selbständige, DIW aktuell, Nr. 47, Berlin.
Schröder, C., J. Goebel, M. M. Grabka, D. Graeber, M. Kroh, H. Kröger, S. Kühne, S. Liebig, J. Schupp, J. Seebauer und S. Zinn (2020), Erwerbstätige sind vor dem Covid-19-Virus nicht alle gleich, SOEPpapers on Multi-disciplinary Panel Data Research, Nr. 1080, Berlin.
